# Different Stages of Phase Transformation in the Synthesis of Nanocrystalline Sr-Hexaferrite Powder Prepared by a Gaseous Heat Treatment and Re-Calcination Method

**DOI:** 10.3390/nano12213714

**Published:** 2022-10-22

**Authors:** Ramin Dehghan, Seyyed Ali Seyyed Ebrahimi, Zahra Lalegani, Bejan Hamawandi

**Affiliations:** 1 Advanced Magnetic Materials Research Center, School of Metallurgy and Materials, University of Tehran, Tehran 11155 4563, Iran; 2 Department of Applied Physics, KTH Royal Institute of Technology, SE-106 91 Stockholm, Sweden

**Keywords:** strontium hexaferrite, GTR mechanism, phase transformation

## Abstract

In this paper, the phase transformation in a gaseous heat treatment and re-calcination (GTR) process for preparing nanocrystalline Sr-hexaferrite powder using methane (CH_4_) was studied. The process included gaseous heat treatment and subsequent re-calcination. Phase composition of the powder and its physical properties were changed significantly owing to formation of different intermediate phases. Sr-hexaferrite powder was prepared by the conventional route as the precursor. The results were represented in a phase transformation map that showed the intermediate phases and clarified the transformation path during the process. As evidenced by the map, the process had four general stages: decomposition of hexaferrite, reduction of iron oxides to pure iron, re-oxidation of iron, and re-formation of hexaferrite with different properties and structure.

## 1. Introduction

Hard ferrites are the least expensive and most commonly used permanent magnet materials and strontium hexaferrite (SrFe_12_O_19_ or SrO·6Fe_2_O_3_), as an M-type hard ferrite, is a ferrimagnetic ceramic compound which has become common material for permanent magnet applications, such as in automotive and microwave devices, and magneto-optical and perpendicular recording media [1,2,3,4]. Due to the unique biological, thermal, and physical properties of these magnets, their use is increasing day by day [5]. Furthermore, discovering the details of Sr-Fe-O phase diagrams and modifying the thermochemical properties of its components, is another reason there is interest in this type of ferrite [6]. Good resistance to becoming demagnetized (high coercivity), high remanence after magnetization, and high magnetic permeability are their most important properties that enable them to store stronger magnetic fields.

The conventional route for processing normal ferrite powders includes grinding the precursor materials, mixing, sintering and milling. Since lower particle size of the powder can be effective to make much better properties, some advanced production methods, such as co-precipitation [7], sol–gel [8], and sol–gel auto-combustion [9,10] have been used by researchers, although the conventional method remains almost the simplest and cheapest one.

The intrinsic magnetic properties of hexaferrites can be controlled and modified by controlling the size, shape, and size distribution of the particles, or by using special heat treatment in a reductive gas. Heat treatment of hexaferrites in a reductive gas atmosphere can also modify the morphology of the ferrite [11]. Gas heat treatment is a novel supplementary method which could convert the conventionally synthesized Sr-hexaferrite powder to a nanocrystalline powder and promote its magnetic properties. This treatment can be applied under the static or dynamic atmospheres of a reductive gas like hydrogen, carbon monoxide, and methane.

Fargali et al. [11] studied the phase dynamics of Sr-hexaferrite nanocrystals at different temperatures with a constant flow of hydrogen gas at normal atmospheric pressure. They showed that oxygen is removed from the surface during exposure to hydrogen. They also confirmed an increase in the different phases of Sr-Fe oxides, Fe oxides, and Fe metal by increasing the time of exposure to hydrogen gas. Koohdar et al. [12] also investigated heat treatment of Sr-hexaferrite under hydrogen gas flow at various conditions. The optimum conditions of 850 °C, 60 cm^3^/min gas flow, and 1 h were obtained for hydrogen treatment. They further performed a subsequent re-calcination to obtain improved magnetic properties. They could obtain 30% increase in the coercivity of re-formed Sr-hexaferrite powder. Bilovol and Martínez-García [13] performed heat treatment under oxygen flow and showed that O_2_ gas prevents the formation of Sr^2+^ ion anchors, in the form of carbonate, and reduces the temperature required to obtain Sr hexagonal ferrite.

Oxides containing iron in two oxidation states are affected not only by temperature, but also by the composition of the atmosphere. Therefore, studies of the interaction of ferrite with the gas phase are very important to understand the nature of material [14].

The present study is a comprehensive study on the phase transformation of Sr-hexaferrite under heat treatment with a dynamic CH_4_ atmosphere and then its re-calcination. Successful use of CH_4_ promises the possibility of using the natural gas to improve economical aspects of the process. The effect of this process (gas treatment and re-calcination) on the magnetic properties, particularly on enhancement of the coercivity of the initial sample, has been reported elsewhere [15].

## 2. Experimental

### 2.1. Materials and Instrumentation

Hematite (α-Fe_2_O_3_, 99%, M = 159.69 g/mol, Merck KGaA, Darmstadt, Germany) and strontium carbonate (SrCO_3_, 99%, M = 147.63 g/mol, Merck) powder were used as precursors to process the primary strontium hexaferrite (SrFe_12_O_19_) powder. Methane gas (CH_4_, 99.99%) was used as a dynamic flow for gaseous heat treatment.

X-ray diffraction analysis (XRD, Philips Co., Pw-1730, Eindhoven, Netherlands) was performed to characterize the phase composition of the products using Cu Kα radiation (λ = 1.54 Å), voltage 40 kV, step size 0.02 degrees, step time 0.25 s and 2θ range from 20° to 100°. The multiphase Rietveld phase quantification method was performed, using MAUD software, to estimate the weight percent of components.

### 2.2. Processing and Re-Forming the SrFe_12_O_19_ Powder

Processing of the primary SrFe_12_O_19_ powder was initiated conventionally by mixing Fe_2_O_3_ and SrCO_3_ powders. The resultant mixture was calcined at 1100 °C for an hour in the air. The next stage was gaseous (CH_4_) heat treatment of the primary SrFe_12_O_19_. The gaseous heat treatment was carried out in a tube furnace with a quartz reactor under a dynamic CH_4_ atmosphere with a flow rate of 30 cc/min for an hour at different temperatures (450, 550, 650, 750, 850, 950, and 1050 °C). The decomposition of SrFe_12_O_19_ commenced until complete reduction of intermediate oxides and formation of pure iron (Fe).

The subsequent re-calcination process to re-form SrFe_12_O_19_ was performed through heating up to different temperatures (200, 300, 400, 500, 600, 700, 800, 900, 1000, 1100, and 1200 °C) and for different times in a muffle furnace with the heating and cooling rates of 10 °C/min.

### 2.3. Calculating the Molar Percent of Components

In order to compare the heat-treated samples, and trace what happens to the Fe atoms in each step, the results were normalized on the basis of the assumption that the initial amount of Fe in the initial powder was 100 moles. The molar percent of each component in all samples was calculated by dividing their weight percent by their molecular weight:

%wt_i_ /MW_i_ = MF_i_

%M_i_ = 100 × MF_i_

where %wt_i_, MW_i_, MF_i_, and %M_i_ are weight percent, molecular weight, molar fraction, and molar percent of the component i, respectively.

The amount of Fe atoms existing in the chemical composition of each component in the samples was calculated using its stoichiometric ratio:

Fe_i_ = %M_i_ × n_i_

where Fe_i_ and n_i_ are the amount of Fe atoms existing in the chemical composition of component i and stoichiometric number of Fe in component i, respectively.

The molar percent of each component in all samples was calculated, assuming the total amount of Fe existing in each sample was 100 units (normalized molar percent). This assumption was possible because all existing components in all samples estimated by the multiphase Rietveld phase quantification method had Fe in their composition. It was a very helpful assumption.

Fe_tot_ = Fe_i_ + Fe_j_ +…+ Fe_n_

%NM_i_ = %M_i_ × 100/Fe_tot_

where Fe_tot_ is the total amount of Fe atoms existing in the sample, Fe_j_ and Fe_n_ are amounts of Fe atoms existing in the chemical composition of components j and n, and %NM_i_ is normalized molar percent of component i.

## 3. Results and Discussion

### 3.1. Decomposition of SrFe_12_O_19_

#### 3.1.1. The Effect of Different Parameters on SrFe_12_O_19_ Decomposition with CH_4_ Gaseous Heat Treatment

**Figure 1:**
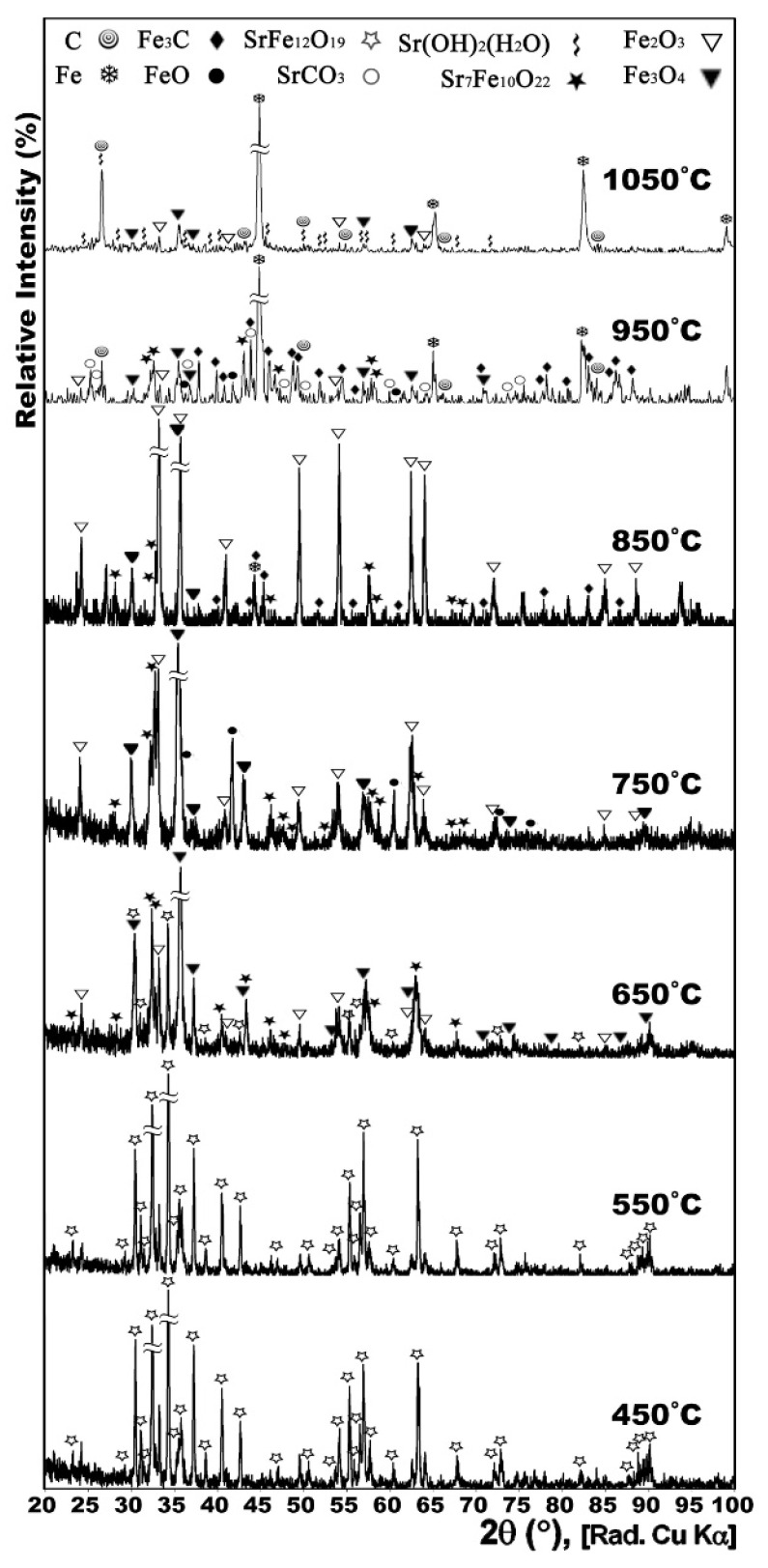
XRD patterns of conventionally synthesized Sr-hexaferrite powders gas heat treated at 450, 550, 650, 750, 850, 950 and 1050 °C for 1 h.

Figure 1 shows the XRD patterns of the conventionally synthesized Sr-hexaferrite along with the XRD patterns of the powders heat treated at 450, 550, 650, 750, 850, 950, and 1050 °C with CH_4_ gas flow of 30 cc/min for 1 h. It can be seen that the decomposition of the hexaferrite started above 550 °C.

Equation (1) shows the decomposition reaction of CH_4_ gas. It shows that in such process conditions, decomposition of methane could be performed at above 550 °C. So, it could be concluded that methane itself was not responsible for the decomposition of ferrite and the reduction might be conducted by hydrogen and carbon; however, hydrogen was more effective.

CH_4_ = C + 2H_2_   ΔG_0_ = 69120 − 22.25TLnT + 65.34T (J) (1)

According to Figure 1, Sr-hexaferrite was partially decomposed to Sr_7_Fe_10_O_22_ and hematite (Fe_2_O_3_), at 650 °C, and, then, the Fe_2_O_3_ was partially reduced to form magnetite (Fe_3_O_4_). At 750 °C, Sr-hexaferrite was completely decomposed and no trace of it could be seen in the XRD pattern. Sr_7_Fe_10_O_22_ seemed to be fully stable but the reduction of Fe_2_O_3_ progressed till the formation of FeO. The formation of Fe from reduction of iron oxides could be observed for the first time at 850 °C; however, cementite (Fe_3_C) was also formed, concurrently. According to a study of the phase dynamics of Sr-hexaferrite under a constant flow of hydrogen gas, nanocrystals that decreased at lower temperatures formed Sr-Fe oxides, such as Sr_7_Fe_10_O_22_, and iron oxides, such as Fe_3_O_4_ and FeO. The researchers introduced the reduction of Sr-hexaferrite nanocrystals in three stages of surface oxygen removal, surface reduction and bulk reduction with activation energies of 55.5, 40.2, and 44.1 kJ mol^−1^, respectively, thus showing that oxygen desorption is a function of a controlled chemical reaction mechanism and surface, and bulk reduction are a function of the two controlled mechanisms of chemical reaction and gas penetration [11].

It is clear that the source of carbon was decomposition of CH_4_ gas, according to the reaction (1). Anyhow, due to magnetic properties, Fe_3_C was not a desirable phase. This composition was not stable at higher temperatures and, as can be seen in Figure 1, at 950 °C, compared with the results at 850 °C, the Fe traces were invigorated along with debilitation of Fe_3_C traces, and it seemed that Fe had stronger peaks at the expense of the weaker peaks of Fe_3_C. Very few traces of iron oxides, carbon and strontium carbonate could be seen.

At 1050 °C, Fe_3_C was decomposed completely and carbon could be observed instead. 

Since complete decomposition of Sr-hexaferrite and reduction of iron oxides up to pure iron was the main purpose of the gas heat treatment, 950 °C was set as the temperature of treatment. Then, in order to avoid the formation of even small traces of Fe_3_C, other effective parameters, like gas flow rate and time of treatment, were optimized.

**Figure 2:**
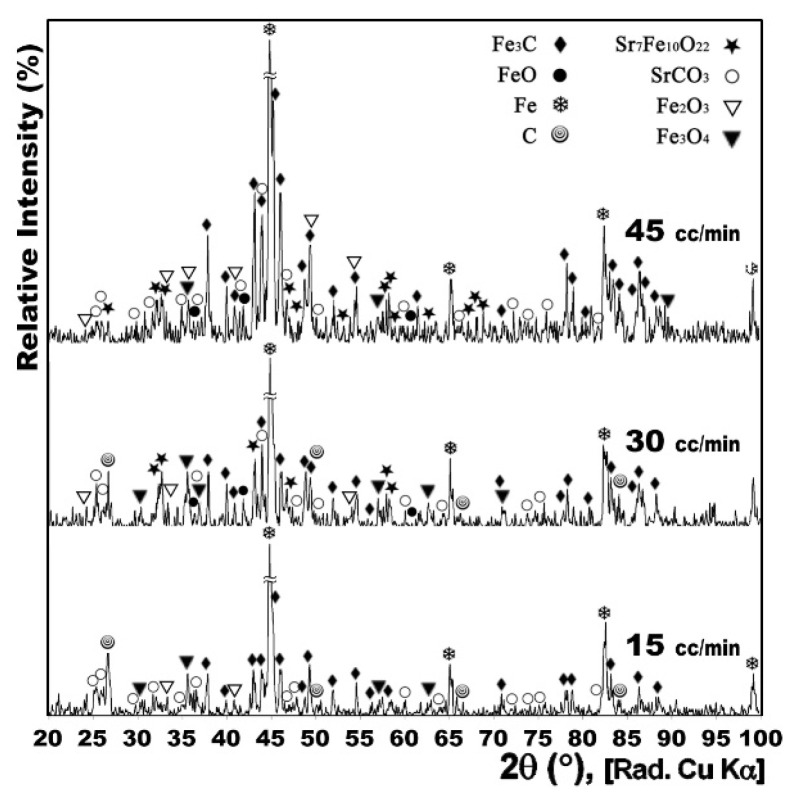
XRD patterns of conventionally synthesized Sr-hexaferrite powders heat treated under dynamic CH_4_ flow of 15, 30 and 45 cc/min at 950 °C for 1 h.

Figure 2 shows the XRD patterns of the powders heat treated at 950 °C with CH_4_ gas flow of 15, 30 and 45 cc/min for 1 h.

When the gas flow decreased from 45 to 15 cc/min it might have enervated the existence of the Fe_3_C phase in the produced powder. Nevertheless, weak traces of Fe_3_C, C, and SrCO_3_ could still be observed in the sample prepared with 15 cc/min gas flow, suggesting that these phases could yet exist in the final product formed during the treatment. In another report [13], SrCO_3_ was reported as a secondary phase of SrFe_12_O_19_ phase transition, and one of the reasons for this was the presence of stress in the SrFe_12_O_19_ crystal lattice. More decrease of gas flow was not applied due to the equipment limitations. Therefore a 15 cc/min rate was selected as the best gas flow.

**Figure 3:**
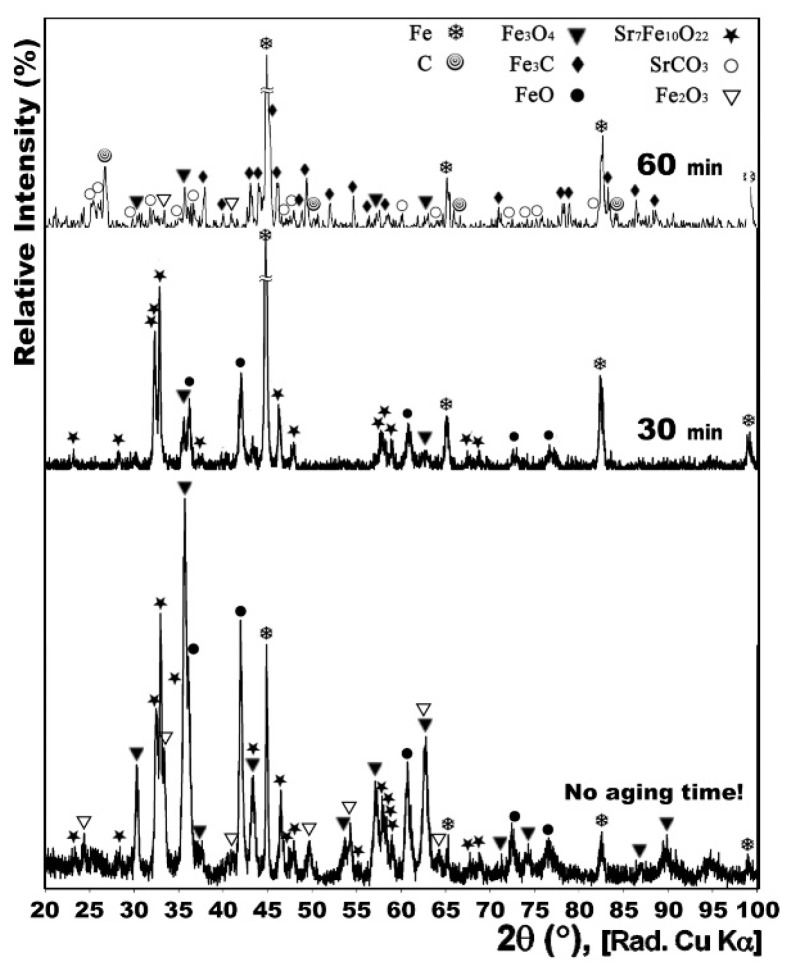
XRD patterns of conventionally synthesized Sr-hexaferrite powders heat treated under dynamic CH_4_ flow of 15 cc/min at 950 °C for 0, 30 and 60 min.

Figure 3 compares the XRD patterns of the powders heat treated at 950 °C with CH_4_ gas flow of 15 cc/min, for 0, 30 and 60 min. Having a 0 min treatment time meant that when the furnace temperature reached 950 °C, the cooling process immediately started.

Both samples treated for 0 and 30 min showed no traces of carbon-containing phases in their XRD patterns. However, while the process was completely performed in the former sample, iron oxides had still not reduced to pure iron in the latter one. Therefore, it could be stated that 30 min duration was not only sufficient, but also necessary, for the heat treatment. Moreover, the existence of the FeO phase in the 0 min treated sample confirmed that the pure Fe formed in the other sample was the product of this intermediate transient phase that itself was formed from reduction of Fe_3_O_4_ during the process.

#### 3.1.2. Investigation of Molar Percent Changes of Components during the SrFe_12_O_19_ Decomposition Process

**Figure 4:**
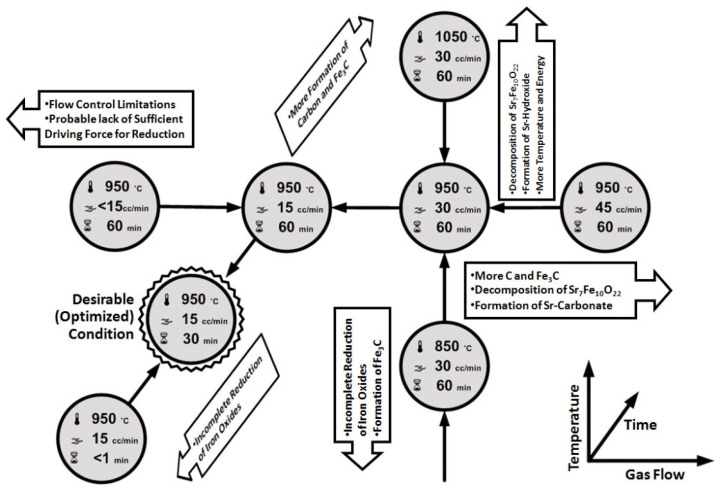
Schematic optimization plan to achieve the best product from gas heat treatment.

It could be concluded that the best condition for the process was 0.5 h heat treatment at 950 °C with CH_4_ gas flow of 15 cc/min. The summary of all the samples mentioned before, with their process conditions and products, are listed in Table 1. Figure 4 represents a schematic investigation plan to achieve the best condition for the gas heat treatment process.

According to Table 1, and due to the Sr-hexaferrite (SrO.6Fe_2_O_3_) structure, decomposition reaction could be generally written as Equation (2):

(2) $$7{\mathrm{SrFe}}_{12}O_{19}\;\overset{H_{2}}{\to }\;{\mathrm{Sr}}_{7}{\mathrm{Fe}}_{10}O_{22}+37{\mathrm{Fe}}_{2}O_{3}$$

This reaction was followed by a complete reduction of hematite to magnetite, wustite, and, finally, pure Fe. SrFe_12_O_19_ as a packing of oxygen ions that had two types of blocks (S and R) with different symmetries. The R-block had a hexagonal structure and three types of interstitial sites (octahedral, tetrahedral, and bipyramidal) containing Fe^3+^ ions. The other block, the S-block, had a spinel structure with cubic symmetry. Sr-hexaferrite heating caused iron ions to diffuse from interstitial sites with lower symmetry to sites with higher symmetry. Such diffusion deformed the R-block, causing the Sr^2+^ ion to move freely to form another environment. On the other hand, during heating, the oxidation state of iron ions changed by a reduction of Fe^3+^ to Fe^2+^. This change in the ionic radius of iron affected the diffusion process and could change the structure from hexagonal to cubic. This was the reason for the formation of iron oxide with cubic symmetry during heating [13]. Although many transient reactions played a role during the process, at the end of treatment the whole reaction could be generally summarized by Equation (3).

7SrFe_12_O_19_ + 111H_2_ = Sr_7_Fe_10_O_22_ + 74Fe + 111H_2_O (3)

**Figure 5:**
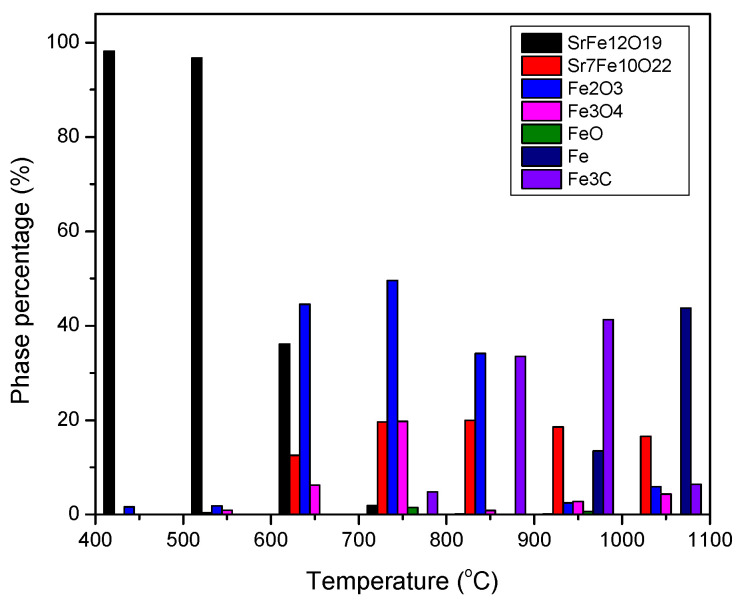
Phase percentage of different phases in the samples obtained by CH_4_ heat treatment at different temperatures.

**Figure 6:**
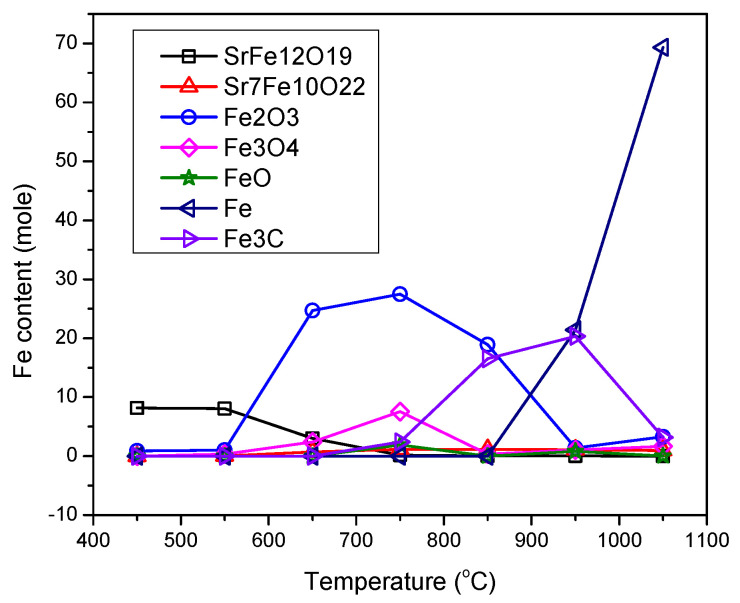
Amount of Fe content in different phases in the samples obtained by CH_4_ heat treatment at different temperatures (normalized on the basis of supposing that the initial amount of Fe in the sample was 100 moles).

The weight percent of components for the samples prepared at different temperatures (Figure 1) were estimated from multiphase Rietveld phase quantification method, using MAUD software. The phase percentages of the different phases are shown in Figure 5 and Table S1. In order to trace what would happen to Fe atoms in each step, the results were normalized on the basis of the supposition that assumes the initial amount of Fe in the initial powder was 100 moles. The results are presented in Figure 6. In this figure, only the phases containing Fe are presented and “normalization for 100 moles Fe” meant that the process had started with an amount of initial powder which contained 100 moles Fe (about 8.33 moles Sr-hexaferrite) and the total amount of Fe in all phases in each step was constant (the same 100 moles). The data related to Figure 6 are also presented in Table S2.

According to Figure 5 and Figure 6, the product of treatment at 450 and 550 °C consisted of about 8 moles (>96%) single phase Sr-hexaferrite. In other words, the whole assumed 100 moles Fe was almost within the hexaferrite composition.

At 650 °C, the amount of Sr-hexaferrite phase reduced to about 3 moles (36%), which meant that more than 60 percent of the initial powder decomposed. In return, about 0.7 moles (13%) Sr_7_Fe_10_O_22_, 24.7 moles (45%) hematite and 2.4 moles (6%) magnetite formed. In other words, 62 moles (>60%) Fe were extracted from partial decomposition of Sr-hexaferrite, which formed Sr_7_Fe_10_O_22_ and hematite. Then, a partial amount of hematite was immediately spent to form the magnetite phase.

At 750 °C only an inconsiderable amount of hexaferrite could be found in the product, indicating that almost all of the initial powder had decomposed, and, in its place, Sr_7_Fe_10_O_22_ reached a high quantity. Figure 6 shows that the amount of this phase was about 1.14 moles, which was approximately a seventh of the initial Sr-hexaferrite. Other research has shown that during heat treatment with hydrogen, SrFe_12_O_19_ decomposes into a mixture of Sr_7_Fe_10_O_22_ and Fe [16,17]. According to research investigating the quasi-binary SrO-Fe_2_O_3_ system, intermediate phases of Sr_n+1_Fe_n_O_3n+1_ (n = 1, 2, 3, ∞), Sr_n_Fe_n_O_3n-1_ (n = 2, 4, 8, ∞), SrFeO_3-δ_, and Sr_7_Fe_10_O_22_, occur [18,19,20,21,22,23]. Some researchers have suggested that oxides with a Sr to Fe ratio greater than one may have non-stoichiometric oxygen and show a mixture of valences Sr^3+^ and Sr^4+^. According to this result, given that Fe/Sr = 1.43 for Sr_7_Fe_10_O_22_ phase, they stated that this phase did not exist and was, in fact, a mixture of two close phases in the ternary diagram, namely SrFeO_x_ and Sr_4_Fe_6_O_13_ [14]. The ternary diagram of the Sr-Fe-O system is shown in ref. [14]. However, in the present study, we showed that a stable compound of Sr_7_Fe_10_O_22_ formed during heat treatment with methane gas.

According to Equation (2), as the first step of the process, and accepting that all hematite, magnetite, wustite, and cementite phases were formed from reduction of hematite afterwards, it could be concluded that, in the beginning, about 43.3 moles of hematite formed, which was nearly 37 times more than that of Sr_7_Fe_10_O_22_. This was in good agreement with the stochiometric coefficients in Equation (2). The following equation shows the conversion of hematite to magnetite and, also, the simultaneous existence of these two phases:

Fe_2_O_3_ ↔ 2/3 Fe_3_O_4_ + 1/6 O_2_ (4)

Subsequent oxygen removal caused a series of transformations in iron oxide and the decomposition of magnetite to wustite [6]:

Fe_3_O_4_ ↔ 3/(1 − n) Fe_1-n_O + (2 − 1.5/(1 − n)) O_2_ (5)

Figure 6 shows that at 1050 °C, due to decomposition of Fe_3_C, about 70 moles of pure iron were produced (which was a 70% total of Fe). In this sample, the amount of Sr_7_Fe_10_O_22_ reduced as a result of its decomposition into strontium hydroxide, as mentioned before (since the latter phase did not contain Fe, it was not presented in Figure 6). It should be noted that the slight observable decrease in the amount of Sr_7_Fe_10_O_22_ phase at 950 °C may have also been related to formation of strontium carbonate.

#### 3.1.3. Phase Transformation Map of SrFe_12_O_19_ Decomposition

**Figure 7:**
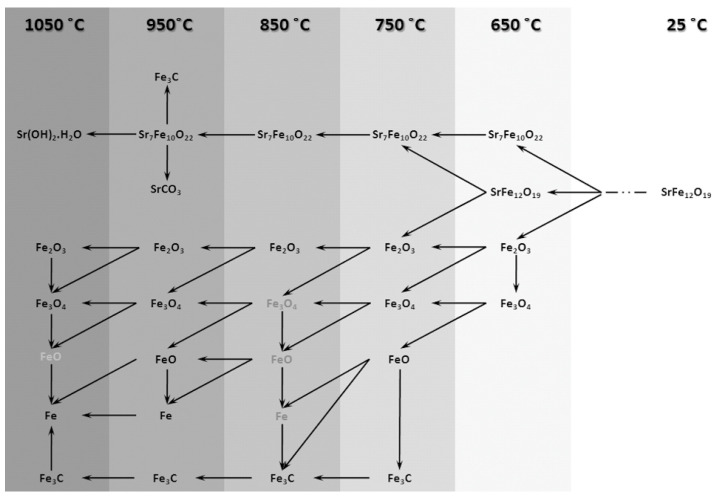
Phase transformation map suggested for the gas heat treatment of Sr-hexaferrite.

Figure 7 suggests a phase transformation map for the CH_4_ gas heat treatment of Sr-hexaferrite revealing the transformation mechanism of the process. In this figure, phases that are shown in grey were the phases that were not detected in the related XRD pattern but they could, anyhow, be considered as transient phases.

### 3.2. Re-Formation of SrFe_12_O_19_ with Re-Calcination Process

#### 3.2.1. The Effect of Different Calcination Parameters on SrFe_12_O_19_ Re-Formation

**Figure 8:**
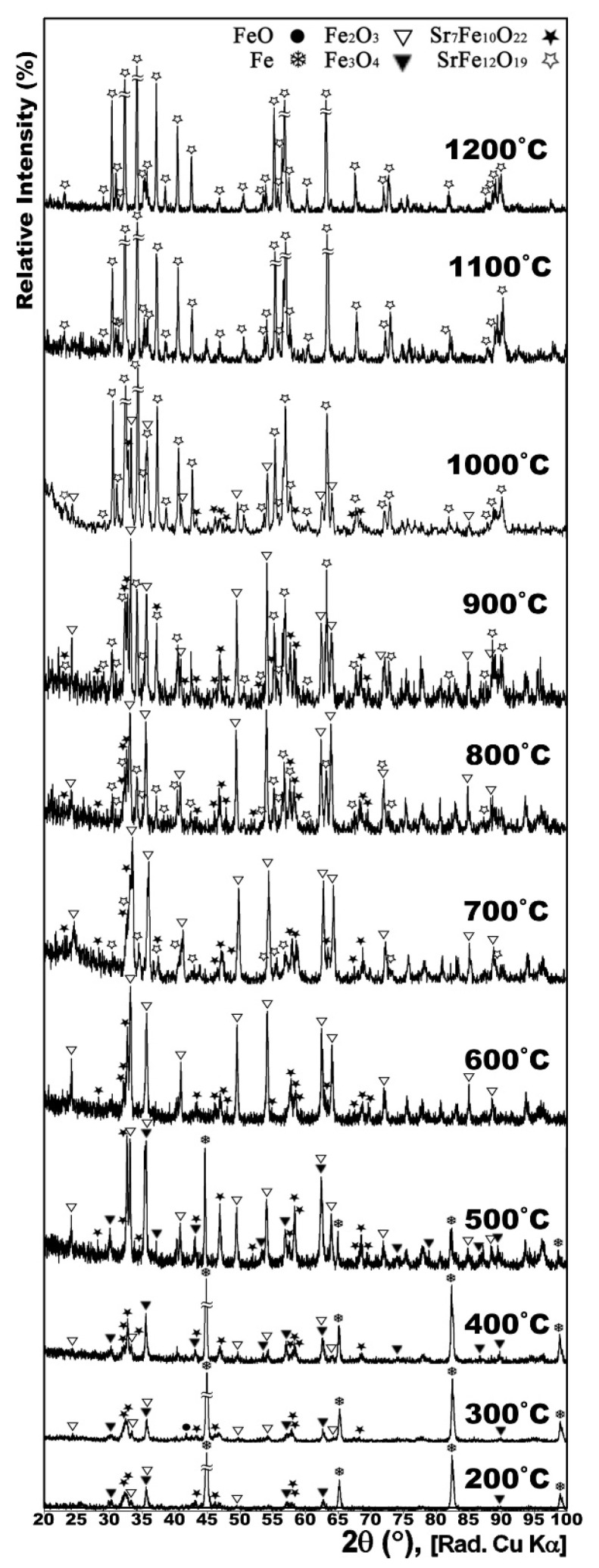
XRD patterns of the CH_4_ heat treated powder, calcined at 200, 300, 400, 500, 600, 700, 800, 900, 1000, 1100 and 1200 °C for 1 h.

As described in the experimental section, in order to form the final product, the gas heat treated powder should undergo a subsequent re-calcination process. Figure 8 shows the XRD patterns of the Sr-hexaferrite heat treated at the best condition (950 °C with CH_4_ gas flow of 15 cc/min for 0.5 h) and re-calcined at different calcination temperatures, of 200, 300, 400, 500, 600, 700, 800, 900, 1000, 1100 and 1200 °C, for 1 h.

The XRD patterns of the samples re-calcined at 200, 300 and 400 °C (Figure 8) were almost the same and were also more or less similar to that of the gas heat treated sample before re-calcination (the middle pattern in Figure 3). This signified that, except for very slight probable oxidation of iron or its oxides, no serious events (such as transformation) happened to the sample up to 400 °C, and Sr_7_Fe_10_O_22_ was also stable.

At 500 °C the arrangement of phases changed. Very strong peaks of Fe in the previous patterns were comparable to the peaks of hematite and magnetite in this pattern. This meant that parts of pure iron engaged in the oxidation into wustite and, consequently, into magnetite and hematite (the absence of the wustite peaks in the pattern could be due to very fast transformation of this phase into upper-level oxides).

The only phases that could be observed at 600 °C were hematite and Sr_7_Fe_10_O_22_. Therefore, Fe and all its oxides had been transformed to the most stable iron oxide, hematite, while Sr_7_Fe_10_O_22_ was also still stable. The condition was the same at 700 and 800 °C, except that, in those patterns, very slight signs of Sr-hexaferrite presence could be considered. 

At 900 °C, the presence of Sr-hexaferrite, along with the two previous phases, was just observable. At 1000 °C, Sr-hexaferrite peaks were much more intensive than those for the other phases; however, the other two phases still had observable peaks. Furthermore, as mentioned in the previous study by our group [15], the TEM micrograph of the CH_4_ heat treated powder calcined at 1000 °C for 1 h showed particles with a size less than 50 nm. 

XRD patterns of the samples calcined at 1100 °C and 1200 °C, respectively, both indicated single phase Sr-hexaferrite. Therefore, it could be concluded that increasing the temperature above 1100 °C was not only unnecessary, but could also probably cause the Sr-hexaferrite crystallites to grow. 

**Figure 9:**
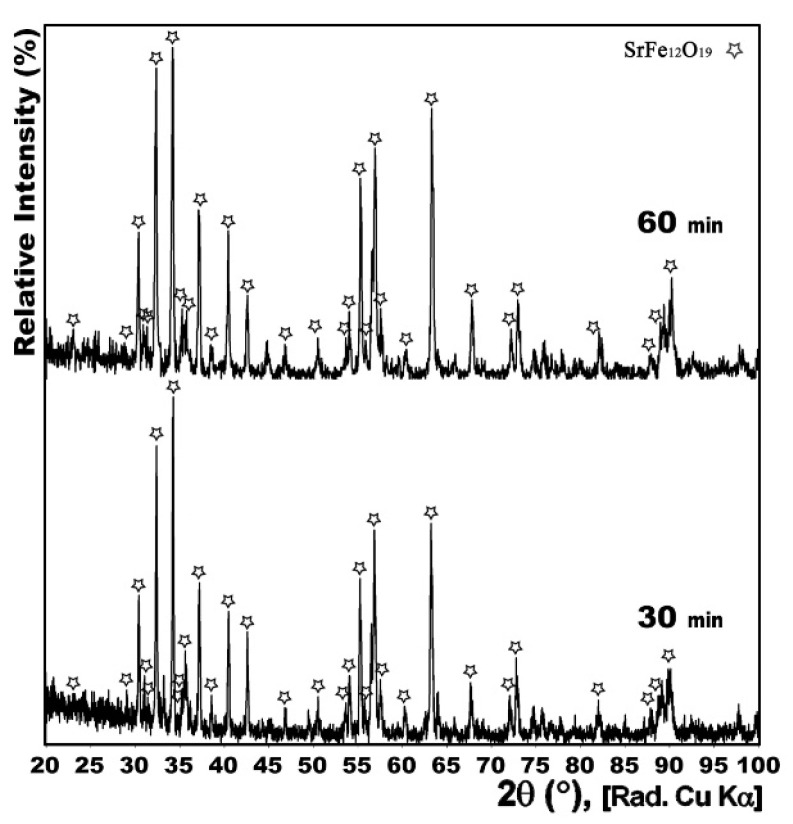
XRD patterns of the CH_4_ heat treated powder calcined at 1100 °C for 0.5 and 1 h.

Since it could be probable to obtain the single-phase Sr-hexaferrite in a shorter time, another sample was prepared by calcination at 1100 °C for only 30 min, rather than for one hour. The result was satisfactory, and, as is shown in Figure 9, the XRD pattern of the sample confirmed that the product, as for the other sample calcined for 1 h, contained single phase Sr-hexaferrite.

Different conditions of the re-calcination process are summarized in Table 2.

Contrary to what was described for the gas heat treatment reactions, here the reactions started with oxidation of pure iron to wustite, and, thereafter, magnetite and then hematite. Finally, re-composition of hematite and Sr_7_Fe_10_O_22_ led to re-formation of Sr-hexaferrite. The whole reaction might be generally written as a reversed version of Equation (3).

**Figure 10:**
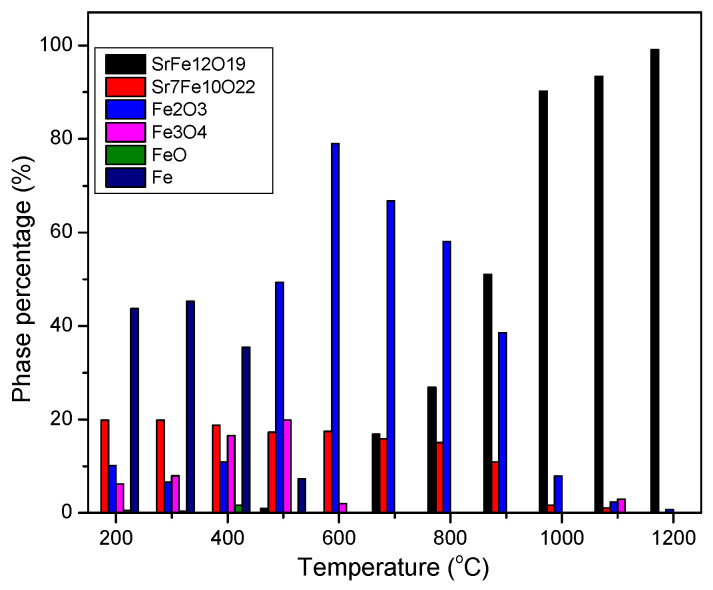
Phase percentages in the gas heat treated samples, re-calcined at different temperatures.

**Figure 11:**
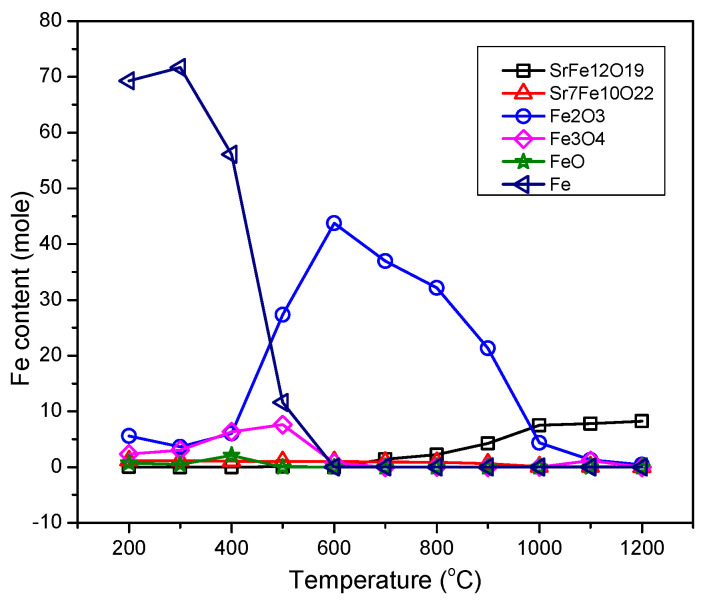
Fe content of different phases in the gas heat treated samples, re-calcined at different temperatures (normalized on the basis of supposing that the initial amount of Fe in the sample was 100 moles).

The weight percent of different phases in the produced samples are presented in Figure 9 and were estimated in Figure 10 and Table S3. The results were normalized again for 100 moles of Fe, presented in Figure 11 and Table S4.

According to Figure 11 a gradual elimination of pure iron occurred between 400 to 600 °C, along with a gradual formation of hematite in coincidence with the elimination of pure iron between 400 to 700 °C, and, then, gradual its elimination between 700 to 1200 °C in a bell curve manner. The re-formation of Sr-hexaferrite started from 700 °C. Researchers have shown that hexagonal ferrite formation reaches its maximum above 780 °C [24]. Emission of methane gas from the decomposition of organic material causes it to recombine with Sr^2+^, and the carbonate decomposes at 700 °C, while the Sr^2+^ ion is able to diffuse to form hexagonal ferrite [13]. Sr_7_Fe_10_O_22_ is stable up to 900 °C and elimination of it occurs at higher temperatures.

#### 3.2.2. Phase Transformation Map of SrFe_12_O_19_ Re-Formation

**Figure 12:**
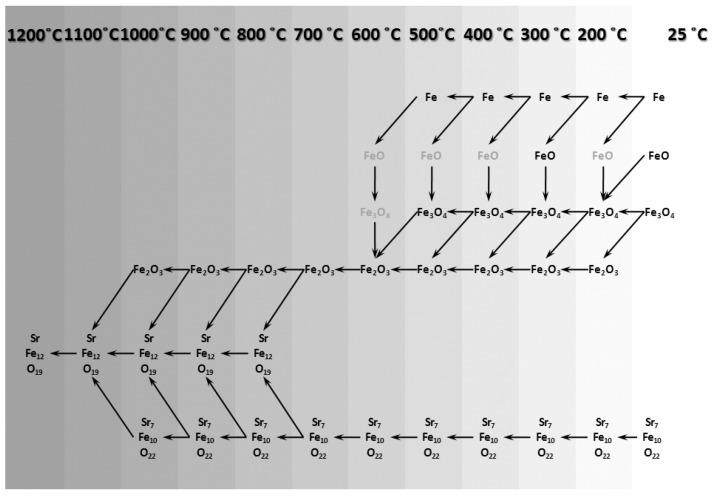
Phase transformation map suggested for the re-calcination of gas heat treated Sr-hexaferrite.

Similar to what was shown in Figure 7, a phase transformation map for the re-calcination process is presented in Figure 12. This figure may reveal the transformation mechanism of re-calcination process. Phases shown in grey are the phases which were not detected in the related XRD pattern but they could, anyhow, be considered as transient phases.

### 3.3. Phase Transformation Map of the GTR Process

**Figure 13:**
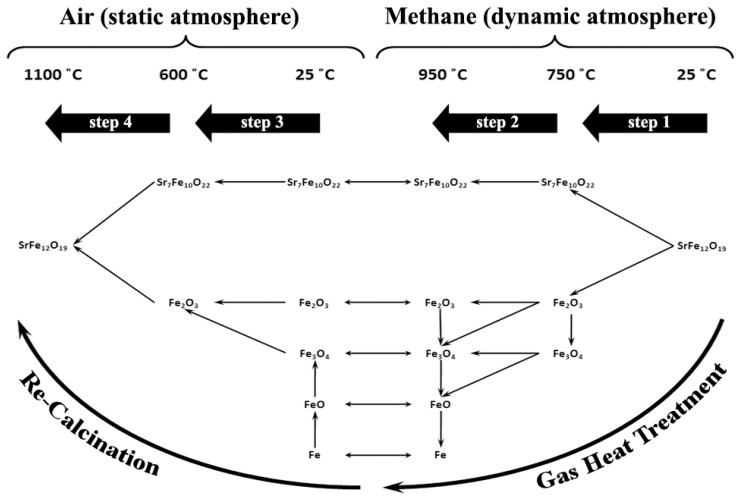
Suggested phase transformation map for the GTR Process of Sr-hexaferrite.

Combining the two Figure 7 and Figure 12 makes it possible to extract the total phase transformation map of the whole GTR process, which is presented in Figure 13. The model presented in this figure, from a phase transformation point of view, consists of four principal stages as follows:

1. Decomposition of strontium hexaferrite
2. Reduction of iron oxides
3. Re-oxidation of iron
4. Re-formation of strontium hexaferrite

In fact, this model suggests 4 stages for the whole process. The first two stages happen in the gas heat treatment cycle and the other two occur in the re-calcination cycle. On the whole, through these 4 process stages, initial conventionally synthesized strontium hexaferrite powder can become strontium hexaferrite nanocrystalline powder with different magnetic properties and higher usability as well.

## 4. Conclusions

The gaseous heat treatment (under a dynamic atmosphere of methane) and re-calcination process for preparing nanocrystalline Sr-hexaferrite powder from conventionally synthesized strontium hexaferrite was successfully carried out and the as yet unknown phase transformation were well studied.

The model suggested for the phase transformation path of the process consists of four principal stages, including decomposition of strontium hexaferrite (into hematite and Sr_7_Fe_10_O_22_), reduction of iron oxides (up to the obtaining of pure iron), re-oxidation of iron (to form hematite again) and re-formation of strontium hexaferrite (with advanced properties).

Phase composition of the powder and its physical properties changed significantly as a result of formation of different intermediate phases during the process. An important aspect of these phase transformations is the gradual changes in the magnetic nature of the material from hard to soft during gas heat treatment and once more from soft to hard during re-calcination.

## Figures and Tables

**Table 1:** Summary of all samples with different process conditions and their products.

Observed Phases	Time (h)	Gas Flow (cc/min)	Temperature (°C)
SrFe_12_O_19_	1	30	450
SrFe_12_O_19_	1	30	550
SrFe_12_O_19_, Sr_7_Fe_10_O_22_, Fe_2_O_3,_ Fe_3_O_4_	1	30	650
Sr_7_Fe_10_O_22_, Fe_2_O_3,_ Fe_3_O_4_, FeO	1	30	750
Sr_7_Fe_10_O_22_, Fe_2_O_3,_ Fe_3_O_4_, Fe_3_C, Fe	1	30	850
Sr_7_Fe_10_O_22_, SrCO_3_, Fe_2_O_3,_ Fe_3_O_4_, FeO, Fe_3_C, Fe, C	1	30	950
Sr(OH)_2_.(H_2_O), Fe_2_O_3,_ Fe_3_O_4_, Fe, C	1	30	1050
Sr_7_Fe_10_O_22_, SrCO_3_, Fe_2_O_3,_ Fe_3_O_4_, FeO, Fe_3_C, Fe	1	45	950
Sr_7_Fe_10_O_22_, SrCO_3_, Fe_2_O_3,_ Fe_3_O_4_, Fe_3_C, Fe, C	1	15	950
Sr_7_Fe_10_O_22_, Fe_3_O_4_, FeO, Fe	0.5	15	950
Sr_7_Fe_10_O_22_, Fe_2_O_3,_ Fe_3_O_4_, FeO, Fe	0	15	950
Sr_7_Fe_10_O_22_, Fe_3_O_4_, FeO, Fe	0.5	15	950

**Table 2:** Summary of all samples with different calcination conditions and their products.

Observed Phases	Time (h)	Calcination Temperature (°C)
Sr_7_Fe_10_O_22_, Fe_2_O_3,_ Fe_3_O_4_, Fe	1	200
Sr_7_Fe_10_O_22_, Fe_2_O_3,_ Fe_3_O_4_, FeO, Fe	1	300
Sr_7_Fe_10_O_22_, Fe_2_O_3,_ Fe_3_O_4_, Fe	1	400
Sr_7_Fe_10_O_22_, Fe_2_O_3,_ Fe_3_O_4_, Fe	1	500
Sr_7_Fe_10_O_22_, Fe_2_O_3_	1	600
SrFe_12_O_19_, Sr_7_Fe_10_O_22_, Fe_2_O_3_	1	700
SrFe_12_O_19_, Sr_7_Fe_10_O_22_, Fe_2_O_3_	1	800
SrFe_12_O_19_, Sr_7_Fe_10_O_22_, Fe_2_O_3_	1	900
SrFe_12_O_19_, Sr_7_Fe_10_O_22_, Fe_2_O_3_	1	1000
SrFe_12_O_19_	1	1100
SrFe_12_O_19_	1	1200
SrFe_12_O_19_	0.5	1100
SrFe_12_O_19_	0.5	1100

## Supplementary Materials

The following supporting information can be downloaded at: https://www.mdpi.com/article/10.3390/nano12213714/s1, Table S1: Phase percentages in the samples obtained by CH_4_ heat treatment at different temperatures; Table S2: Amount of different phases in the samples obtained by CH_4_ heat treatment at different temperatures (normalized on the basis of supposing that the initial amount of Fe in the sample has been 100 moles); Table S3: Phase percentage in the samples gas heat treated and re-calcined at different temperatures; Table S4: The amount of different phases in the samples gas heat treated and re-calcined at different temperatures (normalized on the basis of supposing that the initial amount of Fe in the sample has been 100 moles).
