# Supplementary material for: Different Stages of Phase Transformation in the Synthesis of Nanocrystalline Sr-Hexaferrite Powder Prepared by a Gaseous Heat Treatment and Re-Calcination Method
Source: Nanomaterials (Basel). 2022 Oct 22;12(21):3714. doi: 10.3390/nano12213714 (PMC9657408; doi:10.3390/nano12213714)
Supplement: Supplementary file 1 [file nanomaterials-12-03714-s001.zip › nanomaterials-1981125-supplementary.pdf]

Supplementary Material

Different Stages of Phase Transformation in the Synthesis of Nanocrystalline Sr-Hexaferrite Powder Prepared by a Gaseous Heat Treatment and Re-Calcination Method

Ramin Dehghan [1], Seyyed Ali Seyyed Ebrahimi [1,*], Zahra Lalegani [1] and Bejan Hamawandi [2,*]

[1] Advanced Magnetic Materials Research Center, School of Metallurgy and Materials, University of Tehran, Tehran 11155 4563, Iran

[2] Department of Applied Physics, KTH Royal Institute of Technology, SE-106 91 Stockholm, Sweden

* Correspondence: saseyyed@ut.ac.ir (S.A.S.E.); bejan@kth.se (B.H.)

Table S1. Phase percentages in the samples obtained by CH_4 heat treatment at different temperatures

Temperature (°C)	$SrFe_{12}O_{19}$ (mole)	$Sr_7Fe_{10}O_{22}$ (mole)	Fe_2O_3 (mole)	Fe_3O_4 (mole)	FeO (mole)	Fe (mole)	Fe_3C (mole)
450	98.20	0.00	1.60	0.00	0.00	0.00	0.00
550	96.76	0.35	1.86	0.92	0.00	0.00	0.00
650	36.13	12.58	44.56	6.26	0.00	0.00	0.00
750	1.92	19.64	49.62	19.74	1.53	0.00	4.83
850	0.12	19.99	34.13	0.89	0.00	0.00	33.50
950	0.12	18.61	2.49	2.75	0.66	13.50	41.27
1050	0.00	16.54	5.92	4.35	0.01	43.78	6.44

Table S2. Amount of different phases in the samples obtained by CH_4 heat treatment at different temperatures (normalized on the basis of supposing that the initial amount of Fe in the sample has been 100 moles)

Temperature (°C)	$SrFe_{12}O_{19}$ (mole)	$Sr_7Fe_{10}O_{22}$ (mole)	Fe_2O_3 (mole)	Fe_3O_4 (mole)	FeO (mole)	Fe (mole)	Fe_3C (mole)
450	8.18	0.00	0.88	0.00	0.00	0.00	0.00
550	8.06	0.02	1.03	0.35	0.00	0.00	0.00
650	3.01	0.73	24.68	2.39	0.00	0.00	0.00
750	0.16	1.14	27.48	7.54	1.88	0.00	2.38
850	0.01	1.16	18.90	0.34	0.00	0.00	16.50
950	0.01	1.08	1.38	1.05	0.81	21.38	20.33
1050	0.00	0.96	3.28	1.66	0.01	69.33	3.17

Table S3. Phase percentage in the samples gas heat treated and re-calcined at different temperatures

Temperature (°C)	$SrFe_{12}O_{19}$ (mole)	$Sr_7Fe_{10}O_{22}$ (mole)	Fe_2O_3 (mole)	Fe_3O_4 (mole)	FeO (mole)	Fe (mole)
200	0.12	19.81	10.13	6.18	0.60	43.74
300	0.00	19.81	6.55	7.96	0.40	45.26
400	0.00	18.78	10.87	16.52	1.70	35.41
500	0.96	17.23	49.36	19.90	0.00	7.32
600	0.00	17.40	79.03	2.02	0.00	0.00
700	16.81	15.85	66.77	0.00	0.00	0.00
800	26.89	15.00	58.03	0.08	0.00	0.00
900	51.02	10.85	38.55	0.00	0.00	0.00
1000	90.28	1.72	7.90	0.03	0.00	0.00
1100	93.40	1.03	2.35	3.01	0.00	0.00
1200	99.16	0.00	0.76	0.00	0.00	0.00

Table S4. The amount of different phases in the samples gas heat treated and re-calcined at different temperatures (normalized on the basis of supposing that the initial amount of Fe in the sample has been 100 moles)

Temperature (°C)	$SrFe_{12}O_{19}$ (mole)	$Sr_7Fe_{10}O_{22}$ (mole)	Fe_2O_3 (mole)	Fe_3O_4 (mole)	FeO (mole)	Fe (mole)
200	0.01	1.15	5.61	2.36	0.74	69.27
300	0.00	1.15	3.63	3.04	0.49	71.67
400	0.00	1.09	6.02	6.31	2.09	56.08
500	0.08	1.00	27.34	7.60	0.00	11.59
600	0.00	1.01	43.77	0.77	0.00	0.00
700	1.40	0.92	36.98	0.00	0.00	0.01
800	2.24	0.87	32.14	0.03	0.00	0.00
900	4.25	0.63	21.35	0.00	0.00	0.00
1000	7.52	0.10	4.37	0.01	0.00	0.00
1100	7.78	0.06	1.30	1.15	0.00	0.00
1200	8.26	0.00	0.42	0.00	0.00	0.00
